# Primary Tuberculous Abscess of the Spleen in an Immununocompetent Patient Diagnosed by Biochemical Markers and Radiologic Findings

**DOI:** 10.4021/jocmr800w

**Published:** 2012-03-23

**Authors:** Hyung Hun Kim, Seun Ja Park, Moo In Park, Won Moon

**Affiliations:** a Department of Internal Medicine, Kosin University College of Medicine, Busan, Korea

## Abstract

Tuberculous splenic abscess in an immunocompetent patient is extremely rare. We came across a case of middle aged immunocompetent female who presented with abdominal distension, weight loss, and low grade fever. Abdominopelvic computed tomography showed multiple low density lesions in the spleen with ascites. Interferon-gamma release assay was positive and adenosine deaminase level of ascites was significantly high. No primary focus of infection was detected. The patient was diagnosed as having primary multiple tuberculous splenic abscesses with tuberculous peritonitis causing ascites. Follow up computed tomography, after completion of six month course of anti-tubercular therapy, showed splenic abscess and ascites completely disappeared.

**Keywords:**

Spleen; Abscess; Tuberculosis; Immunocompetent; Adenosine deaminase; Interferon-gamma

## Introduction

Splenic abscess is an uncommon disease and tuberculous splenic abscess is much rarer [1-3]. Although tuberculous splenic abscess is increasing in immunocompromised patients due to acquired immunodeficiency syndrome (AIDS), tuberculous abscess in immunocompetent patients is still rare. Moreover, primary tuberculous splenic abscess in an immunocompetent patient is extremely rare. Up to now, only five cases have been reported [3-7]. All these cases were diagnosed histopathologically through splenic biopsy, exploratory laparotomy, and splenectomy [3-7]. In contrast, we experienced one such case diagnosed by abdominopelvic computed tomography and biochemical markers: adenosine deaminase and interferon-gamma release assay. Moreover, the patient completely recovered from tuberculous splenic abscess through anti-tuberculous therapy.

## Case Report

**Figure 1:**
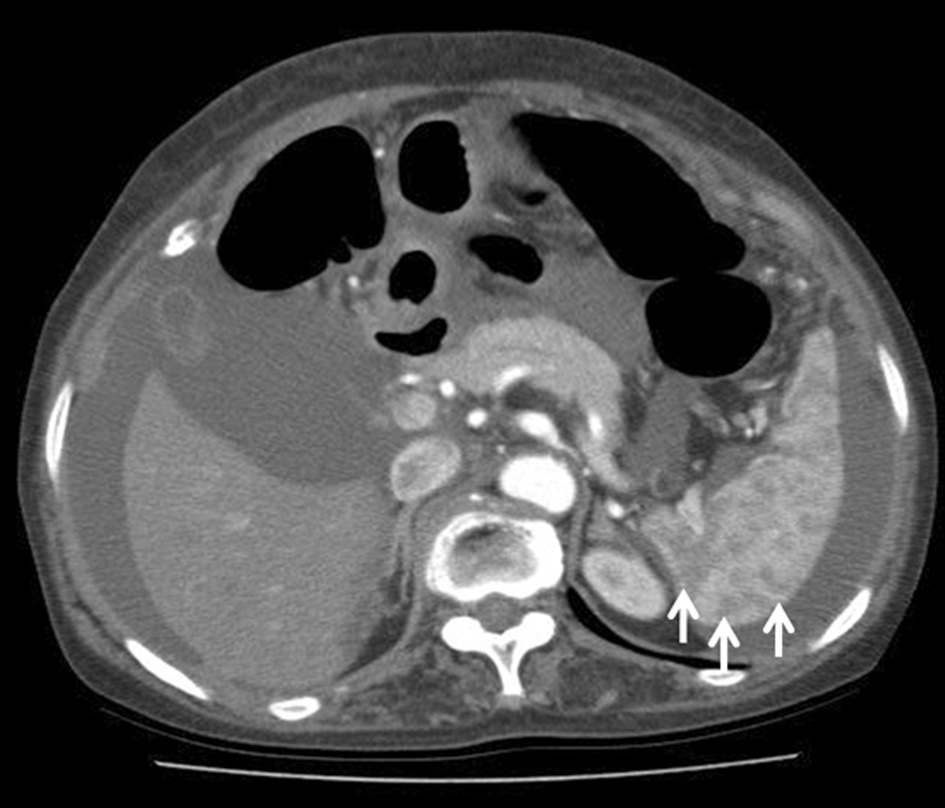
Initial abdominopelvic computed tomography reveals the spleen is occupied with multiple, well-defined, low density, lesions measured 0.5 to 1.5 cm in sized (arrows) and moderate amount of ascites.

**Figure 2:**
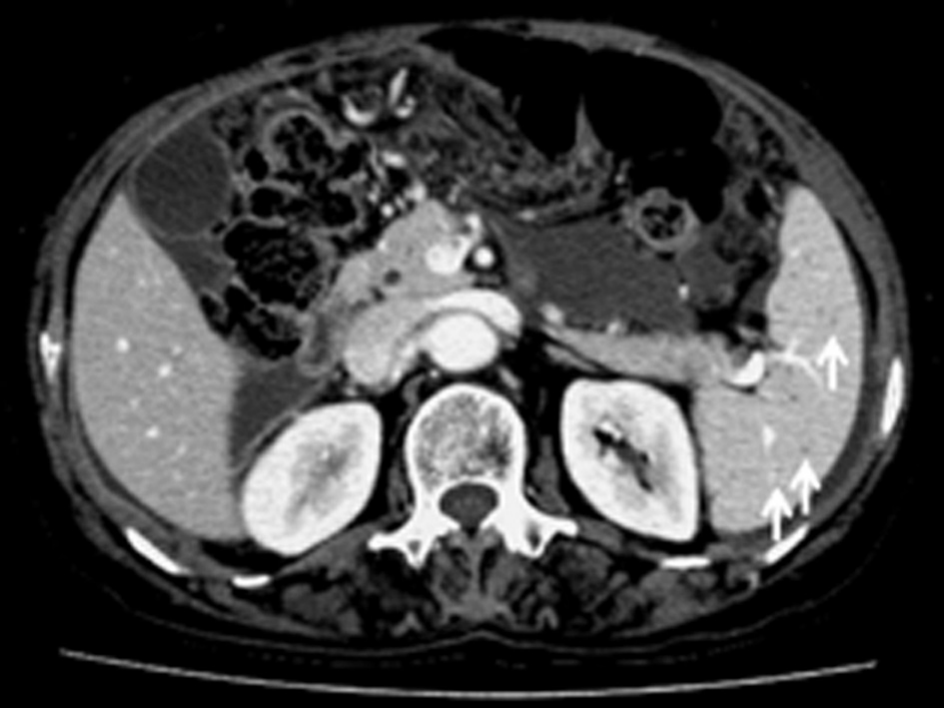
Follow-up computed tomography one month after anti-tubercular therapy, shows low density nodules decreased in size (arrows) with smaller amount of ascites.

**Figure 3:**
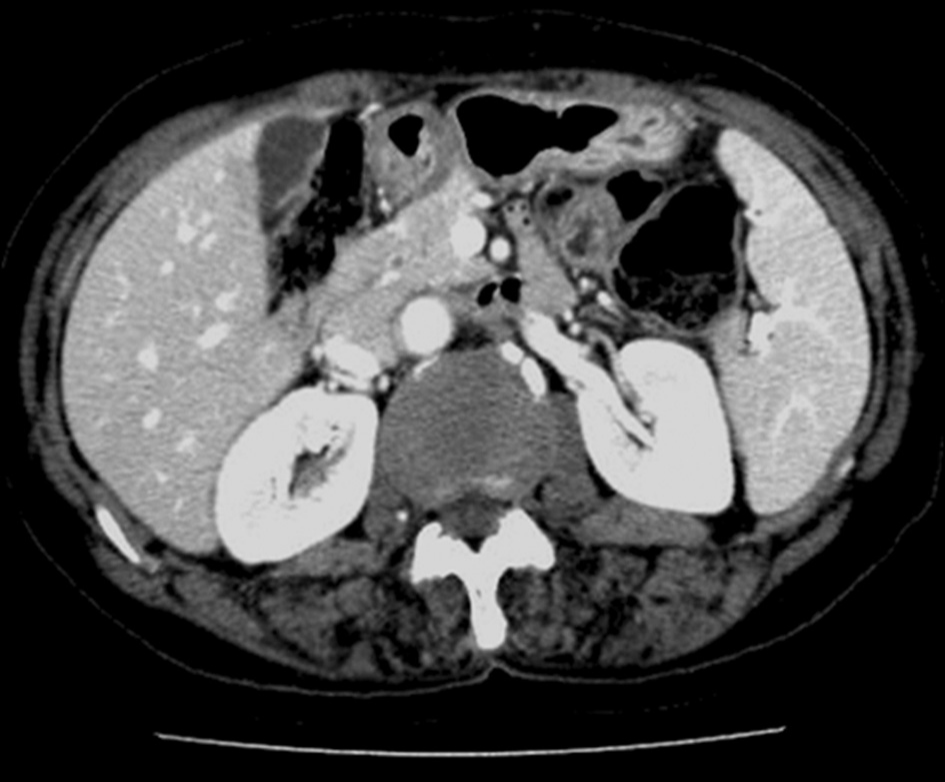
Computed tomography, after six month course of anti-tubercular therapy, demonstrates low density nodules and ascites disappeared completely.

A 55-year-old female patient attended the gastroenterology clinic with two-month history of gradually developed abdominal distention and low-grade febrile sensation. Abdominal distension was associated with dyspepsia and five kilogram-weight loss for last two months. She had taken very small amount of food because she thought eating food would aggravate abdominal distension. There was no past history suggesting hepatitis and other chronic illness such as diabetes mellitus. On physical examination, the patient had low-grade fever (37.8 ^o^C) with low body built and poor nutritional status despite of good socioeconomic status. Abdominal examination revealed shifting dullness and fluid wave. Her routine hematological and biochemical investigations were within normal limits except low serum albumin level (3.0 g/dL). Enzyme-linked immunosorbent assay test for HIV was negative, and her CD4 count was 702/cu mm. Markers for viral hepatitis were negative, and stool examination did not reveal any parasite. Chest X ray was normal. A contrast-enhanced abdominopelvic computed tomography (CT) scan revealed splenomegaly which major axis was approximately 12.5 cm and moderate amount of ascites. The spleen was occupied with multiple, well-defined, low density, lesions measured 0.5 to 1.5 cm in size (Fig. 1). There, however, was no significant intraabdominal lymphadenopathy. Ascites was clear, and notable laboratory data of ascites were followings: white blood cell count, 1,300/mm^3^ (lymphocyte, 90%); albumin, 2.5 g/dL (serum ascites albumin gradient was 0.5 g/dL) and adenosine deaminase (ADA), 76.9 IU/L (normal: up to 45 IU/L). Her serum adenosine deaminase level was 67.5 IU/L (normal: up to 45 IU/L), so ascitic fluid/serum ADA ratio was 1.139. Interferon-gamma release assay (QuantiFERON^®^-TB Gold, Cellestis Limited, Carnegie, Victoria, Australia) was proved to be positive. Repeated cytologic examinations of ascites did not find an abnormal cell. These findings strongly suggested tuberculous peritonitis. Radiologic findings and biochemical tests made the patient diagnosed as having multiple tuberculous splenic abscesses with tuberculous peritonitis. We planned six-month course of oral anti-tubercular regimen: Isoniazide: 300 mg, Rifampin 600 mg, Pirazinamide 1.5 g, Ethambutol 1 g. After two-week of anti-tubercular therapy, the patient’s ascites started to decrease gradually, and she gained strength rapidly. Follow-up CT scan performed one month after treatment showed the size of low density nodules in the spleen markedly decreased, and the amount of ascites was smaller than before (Fig. 2). Six month-follow up CT scan showed low density nodules in the spleen disappeared (Fig. 3), and she recovered completely.

## Discussion

Tuberculous splenic abscess usually is confirmed by involvement of other organs. For this reason, invasive diagnostic modalities often need not to be involved in the workup of splenic lesions in tuberculous patients [8]. Contrary to tuberculous splenic abscesses involving other organs, all reported five cases with primary tuberculous splenic abscesses were diagnosed histopathologically via splenic biopsy, splenic abscess drainage, exploratory laparotomy, and splenectomy after treatment failure. However, we diagnosed primary splenic abscess by radiologic findings and biochemical markers: interferon-gamma assay and ADA. Non-invasive imaging modalities, such as ultrasonography and CT scan, play a role in the assessment of the extent of organ involvement, need for surgical intervention, and therapeutic response [8]. Contrast to these usual usages of image modalities, abdominopelvic CT scan played an imperative role for the diagnosis of splenic tuberculous abscess in our patient because our patient had only splenic lesions and refused percutaneous splenic biopsy. Typical CT findings are multiple, round or ovoid, low density lesions without calcification such as our case. In differential diagnosis of CT findings, lymphoma, hydatid disease, and metastases must be considered [6].

Adding to typical radiologic findings, Interferon-gamma release assay played an important role also. Interferon-gamma release assay is an *in vitro* laboratory diagnostic test using a whole blood specimen. It is an indirect test for *M. tuberculosis* complex (i.e., *M. tuberculosis, M. bovis, M. africanum, M. microti, M.canetti*) infection. The specificity of the test for the low-risk group was 98.1% and the sensitivity for patients with *M. tuberculosis* infection was 89.0% [9].Interferon-gamma release assay was positive in our patient. Another important biochemical marker was ADA in ascites and serum. Levels above 36 IU/L in ascitic fluid (sensitivity: 100%, specificity: 97.1%) and above 54 IU/L in the serum (sensitivity: 81.5%, specificity: 97.6%) suggest tuberculosis. The ascitic fluid/serum ADA ratio over 0.984 was suggestive of tuberculosis (sensitivity: 57.1%, specificity: 87.7%) [10]. Our patient’s ascitic fluid and serum ADA were 76.9 IU/L and 67.5 IU/L respectively. Moreover, ascitic fluid/serum ADA ratio was 1.139. These biochemical tests were quite helpful for us to have confidence that our patient had tuberculous splenic abscess and tuberculous peritonitis.

Because our patient had not received any anti-tubercular therapy previously, we started six-month anti-tubercular therapy, which was effective in our patient. Her ascites started to decrease rapidly after two-week anti-tubercular therapy, and splenic tuberculous abscesses disappeared in three months.

Considering our case, physicians should bear it in mind that primary tuberculous splenic abscess can be diagnosed by typical radiologic findings and biochemical markers such as interferon-gamma release assay and ADA, if there is ascites, without splenic biopsy. Adding to this, starting anti-tubercular regimen will be a reasonable choice for an immunocompetent patient without previous tuberculosis, and the good response to medication will be another indicator to confirm tuberculous splenic abscess.
